# Characterization of lncRNAs involved in cold acclimation of zebrafish ZF4 cells

**DOI:** 10.1371/journal.pone.0195468

**Published:** 2018-04-10

**Authors:** Penglei Jiang, Yanwen Hou, Weikang Fu, Xiaofan Tao, Juntao Luo, Hanxu Lu, Yicheng Xu, Bingshe Han, Junfang Zhang

**Affiliations:** 1 Key Laboratory of Exploration and Utilization of Aquatic Genetic Resources (Shanghai Ocean University), Ministry of Education, Shanghai, China; 2 National Demonstration Center for Experimental Fisheries Science Education, Shanghai Ocean University, Shanghai, China; 3 International Research Center for Marine Biosciences at Shanghai Ocean University, Ministry of Science and Technology, Shanghai, China; Kunming University of Science and Technology, CHINA

## Abstract

Long non-coding RNAs (lncRNAs) are increasingly regarded as a key role in regulating diverse biological processes in various tissues and species. Although the cold responsive lncRNAs have been reported in plants, no data is available on screening and functional prediction of lncRNAs in cold acclimation in fish so far. Here we compared the expression profile of lncRNAs in cold acclimated zebrafish embryonic fibroblast cells (ZF4) cultured at 18°C for 30 days with that of cells cultured at 28°C as control by high-throughput sequencing. Totally 8,363 novel lncRNAs were identified. Including known and novel lncRNAs, there are 347 lncRNAs up-regulated and 342 lncRNAs down-regulated in cold acclimated cells. Among the differentially expressed lncRNAs, 74 and 61 were detected only in control cells or cold-acclimated cells, respectively. The Gene Ontology (GO) and Kyoto Encyclopaedia of Genes and Genomes (KEGG) enrichment analyses of adjacent genes to the differentially expressed lncRNAs showed that the enriched genes are involved in electron transport, cell adhesion, oxidation-reduction process, and so on. We also predicted the target genes of the differentially expressed lncRNAs by looking for interactions between lncRNAs and mRNAs, and constructed an interaction network. In summary, our genome-wide systematic identification and functional prediction of cold responsive lncRNAs in zebrafish cells suggests a crucial role of lincRNAs in cold acclimation in fish.

## Introduction

Long noncoding RNAs (lncRNAs) are generally defined as a class of transcripts with length more than 200 nucleotide (nt) but lack significant protein coding capacity [1]. Accumulating studies suggest that lncRNAs play key roles in regulating development, tumorigenesis, and response to abiotic stresses [1,2]. Unlike the translational regulation mechanism of miRNA is well characterized, mechanism of lncRNAs is still far from a full understanding. They serve as transcriptional silencers, co-activators, and even competing endogenous RNAs by providing interaction sites for miRNAs [3]. lncRNAs also play key roles in regulating gene and genome activity at various levels including serving as ligands or cofactors to mediate histone modification [4] and DNA methylation [5], modulating general transcription factors [6] and RNA pol II loading [7], regulating the activity of transcriptional factors [8].

Fish, one kind of aquatic ectotherm, might face a wide range of temperature variations in its life cycle. Environmental temperature fluctuations are usually accompanied by detectable changes of mRNA and miRNA expression patterns, DNA methylation and histone modification in fish [9–12]. However, the reports about the roles of lncRNA under cold pressure are sparse [2]. In mice, over-expression of TUG1 lncRNA (TUG1, taurine up-regulated gene 1) protects against cold-induced injury of livers by inhibiting apoptosis and inflammation [13]. It is also reported recently that lncRNAs are involved in the response to cold stress in cassava and Chinese cabbage, respectively [14,15]. However these studies are mainly conducted in mammals or plants [2,13–16], how lncRNAs participate in cold acclimation in fish still remains unclear.

Zebrafish is a major model system for study of development, disease and other biological processes. Zebrafish can survive within a wide temperature range of 16.5–38.6°C [17], making zebrafish a good model to study cold acclimation [9–11]. In our preliminary study, we investigated the role of DNA methylation in zebrafish ZF4 cells when experimentally acclimated at 18°C for 30 days, suggesting that DNA methylation is involved in cold acclimation via regulation of genes related to anti-oxidant system, apoptosis, development, chromatin modifying and immune system [11].

In this study, we further studied the roles of lncRNAs in cold acclimation of ZF4 cells. We identified and characterized the lncRNAs responding to cold acclimation and predicted the functions of these lncRNAs in cis and trans. Our data will contribute to better understanding of the roles of lncRNAs in cold acclimation in fishes.

## Materials and methods

### Cell culture and treatment

ZF4 cell line was from the American Type Culture Collection (ATCC, CRL 2050). Cells were cultured in Dulbecco's modified Eagle's medium/F12 nutrient mix (SH30023.01B, Hyclone, Thermo Scientific) supplemented with 10% fetal bovine serum (10099141, Gibco, Life technologies), 1% penicillin-streptomycin-glutamine solution (SV30082.01, Hyclone, Thermo Scientific), at 28°C, 5% CO_2_. For cold acclimation, ZF4 cells were seeded at 40–50% confluence and the next day transferred to an incubator at 18°C, 5% CO_2_, in the same medium for up to 30 days.

### RNA-Seq

Total RNA was extracted using miRNeasy Mini Kit (217004, Qiagen) and purified by RNAClean XP Kit (A63987, Beckman Coulter) and RNase-Free DNase Set (79254, Qiagen). Libraries were constructed using TruSeq Stranded Total RNA LT Sample Prep Kit with Ribo-Zero (RS-122-2301/ RS-122-2302, Illumina). Libraries were pooled and sequenced using the Illumina HiSeq machine as 150-bp paired-end sequencing reads.

### RNA-Seq read alignment and transcript assembly

Clean RNA-Seq reads for each sample were aligned by HISAT2 (2.1.0) with default setting to the zebrafish genome assembly using the Ensembl annotation DanRer10 (Danio_rerio.GRCz10.88.gtf) [18]. Transcripts were assembled by StringTie (1.3.3) with parameter “-G Danio_rerio.GRCz10.88.gtf” [19]. After each sample was assembled, all assemblies were merged together utilizing StringTie’s “merge” function, which merged all the genes found in any of the samples.

### Identification of lncRNAs

After the merging step, gffcompare (0.10.1) was used to compare assembled transcripts and Ensembl annotation DanRer10. To obtain the transcripts that failed to match the known transcripts, five categories of transcripts were extracted, including “Potentially novel isoform”, “A transfrag falling entirely within a reference intron”, “Generic exonic overlap with a reference transcript”, “Unknown, intergenic transcript”, and “Exonic overlap with reference on the opposite strand”. We also compared the residual transcripts with known lncRNAs in the NONCODE2016 database and filter out the same or similar transcripts [20]. The remaining unknown transcripts were filtered by length 200 nt, exon number 1, ORF 300 nt. CPC (Coding Potential Calculator), CNCI (Coding Non-Coding Index) and Pfam were used to delete transcripts with coding potential [21–24]. Finally, we calculated fragments per kilobase per million mapped reads (FPKM) of per transcript and remained transcripts with FPKM > 0.5 at least in one sample as presumed novel lncRNAs. All mRNAs, known lncRNAs and novel lncRNAs were quantified as FPKM by StringTie. Differentially expressed transcripts and genes were determined by edger with a fold change >2 and false discovery rate (FDR) < 0.05.

### Target gene prediction of lncRNAs

All genes within 10 kb of the differentially expressed lncRNAs and nearest genes beyond 10 kb in downstream or upstream were picked out as cis-target genes. The top 20 lncRNAs, with smallest FDR value, were used to predict the trans-regulated genes by RNAplex [25].

### GO and KEGG enrichment analyses

Database for Annotation, Visualization and Integrated Discovery (DAVID) v6.8 web tool (https://david.ncifcrf.gov/) were used to perform GO and KEGG enrichment analyses with a significance of P < 0.05 [26,27].

### Quantitative reverse transcriptase PCR (qRT-PCR)

Total RNA was isolated using TRlzol reagent (15596–026, Life Technologies). Reverse transcription (RT) was performed using 1μg of total RNA with PrimeScript™RT reagent Kit with gDNA Eraser (RR047A, TaKaRa), according to the manufacturer’s instructions. PCR amplification was performed for 2 min at 50°C and 10 min at 95°C, followed by 40 cycles at 95°C for 10 s, and annealing at 60°C for 30 s. Relative mRNA level was analyzed by the comparative CT method. Data were normalized to β-actin. Statistical analysis was performed using GraphPad Prism 5 software. The Student’s T test was used for measurements of gene expression of samples from control group and cold acclimation group from 3 experimental replicates. Primers for qRT-PCR analysis are shown in S1 Table.

## Results

### Identification of novel lncRNAs in ZF4 cells

**Fig 1:**
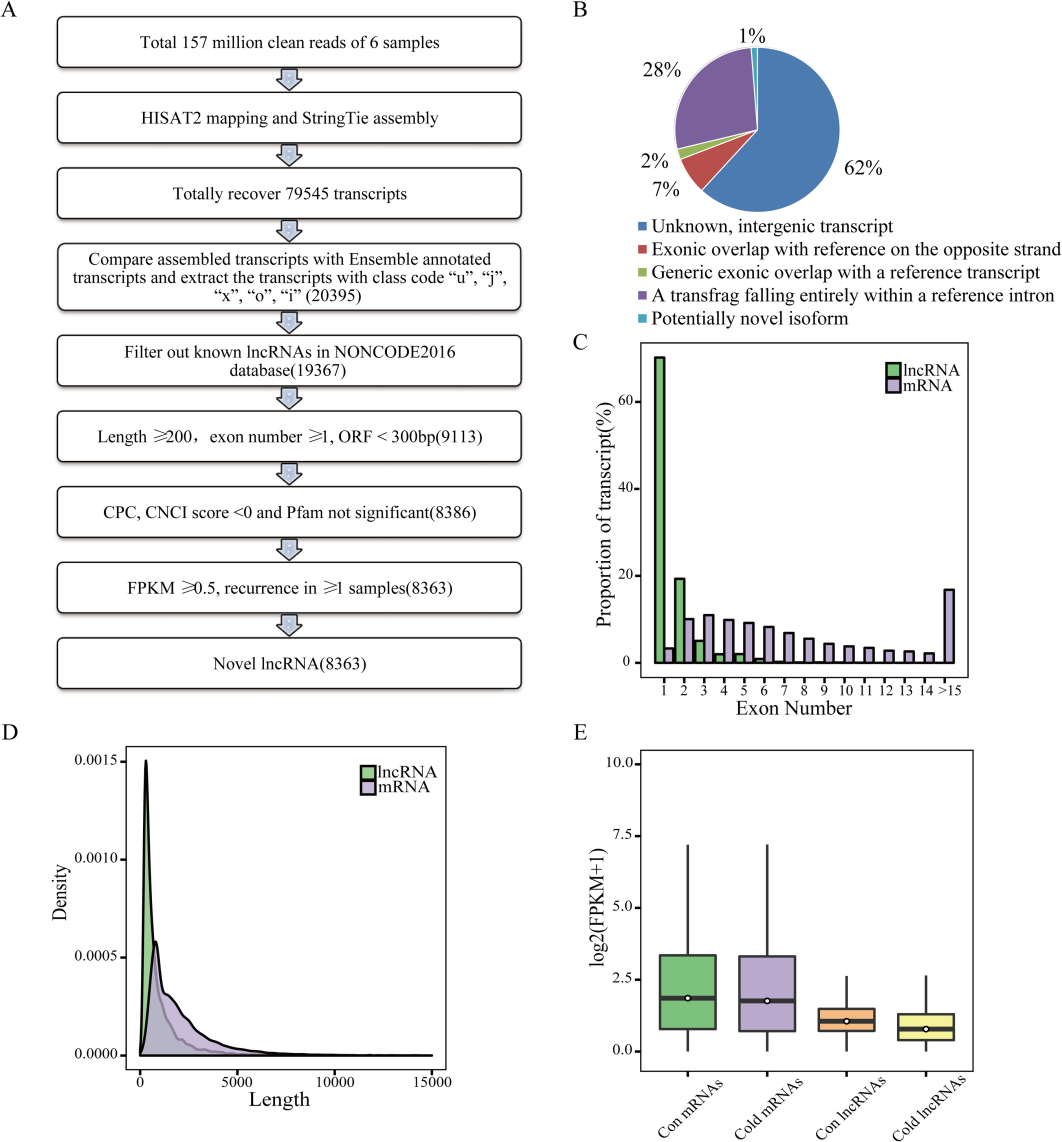
Identification and characterization of lncRNAs. (A) Workflow for identification of lncRNAs. The value in parentheses shows the number of transcripts. (B) Distribution of lncRNA in different chromosomal regions. (C-E) Comparison of exon numbers, transcript lengths, and expression levels between mRNAs and novel lncRNAs. Con: control cells; cold: cold acclimated cells.

Our previous study showed that zebrafish ZF4 cells develop cold acclimation after a 30-day culture at 18°C, a much lower temperature than the normal culture temperature of 28°C [11]. To investigate the roles of non-coding RNAs in this process, total RNAs from cold-acclimated and normal cultured ZF4 cells were subjected to RNA-Seq. After trimming adapters and filtering out low quality reads, more than 157 M clean reads were obtained and nearly 86% could be mapped to the zebrafish genome (danRer10). StringTie, a faster and more efficient assembler than Cufflinks [19], was applied to assemble the transcripts. Total 79,678 transcripts were assembled and 63.3% of them are mRNAs. Following the steps described in Fig 1A, 8,363 presumed novel lncRNAs were discovered at 7,807 loci. Among the novel lncRNAs, about 62% locate at intergenic regions and 27% lie at intron regions (Fig 1B).

We compared the basic genomic features between the novel lncRNAs and known mRNAs. Over 89% novel lncRNAs have no more than 2 exons, while above 86% mRNAs contain no less than 3 exons (Fig 1C). The transcript length of more than 70% lncRNAs, and of only about 33% mRNAs, is less than 1 Kb (Fig 1D). FPKM analysis showed that the expression levels of most lncRNAs are lower than those of mRNAs (Fig 1E). The median FPKM values of lncRNAs and mRNA are 0.9 and 2.6 respectively. In summary, lncRNAs had fewer exons, shorter transcripts, and lower expression than that of mRNAs.

### Identification of differentially expressed lncRNAs (DE-lncRNAs) under cold pressure

**Fig 2:**
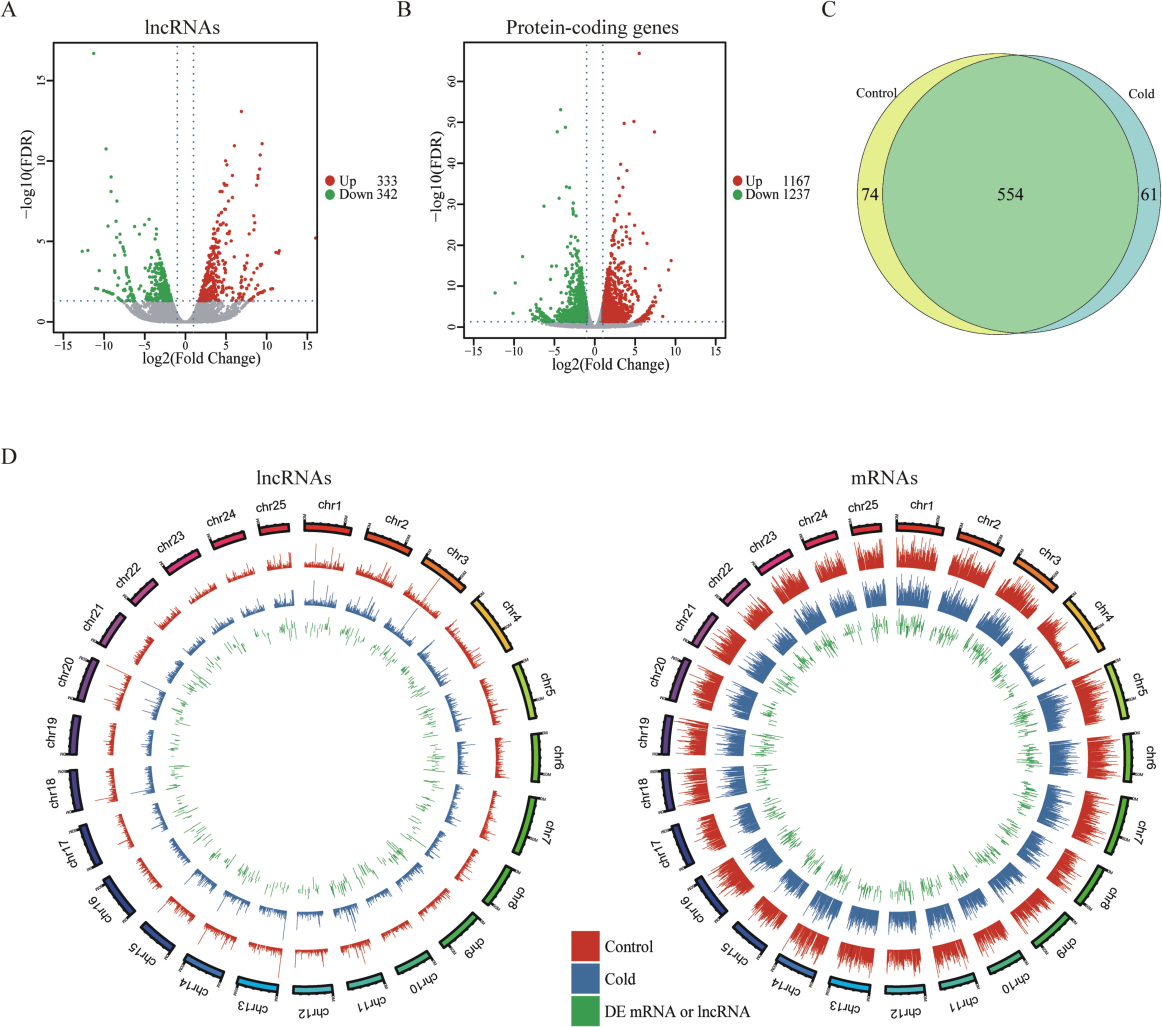
Differential expression patterns of lncRNAs between control and cold acclimated cells. (A-B) The Volcano plots of differentially expressed lncRNAs and protein-coding genes between control or cold acclimated cells, respectively. Abscissa represents log2 (fold-change), and ordinate represents -log10 (FDR). Red dots denote the significantly up-regulated lncRNAs or genes. Green dots denote the significantly down-regulated lncRNAs or genes. Blue dots denote the non-differentially expressed lncRNAs or genes. (C) Venn diagram shows the number of the differentially expressed lncRNAs expressed only in normal cultured or cold acclimated cells. (D) Distribution of mRNAs or lncRNAs along each chromosome. Red and blue represent the log-transformed FPKM values in control or cold acclimated cells, respectively. Green represents the log-transformed fold change, outward and inward bars represent up-regulated and down-regulated RNA, respectively (generated using ggbio R package).

As shown in Fig 1E, both mRNAs and lncRNAs showed decreased expression levels during cold acclimation and this trend is more obvious for lncRNAs, which is consistent with the phenomenon in cassava [14]. Totally 10, 926 lncRNAs and 39, 167 mRNAs were expressed in control cells, while 10, 688 lncRNAs and 39, 338 mRNAs were expressed in cold acclimated cells, the ratio of mRNAs to lncRNAs increased slightly during cold acclimation. The novel lncRNAs and known lncRNAs (NONCODE2016 database) were combined to perform differential expression analysis. The result showed 347 up-regulated and 342 down-regulated lncRNAs after cold acclimation (Fig 2A). Differential expression analysis of protein-coding genes showed 1167 genes up-regulated and 1237 genes down-regulated (Fig 2B). Among the DE-lncRNAs, 74 specifically expressed in control ZF4 cells and 61 solely expressed in cold acclimated ZF4 cells (Fig 2C), indicating a temperature-specific expression pattern for these lncRNAs. According to the visible lncRNA levels in the two groups, lncRNAs are evenly distributed across the 25 chromosomes of zebrafish with no obvious preference for location (Fig 2D). It is interesting that lncRNAs are evenly distributed on the 25 chromosomes without any prejudice expression, while the expression levels of mRNAs on chromosome 4 are remarkably lower (Fig 2D).

### Prediction of cis-target genes of the DE-lncRNAs

**Fig 3:**
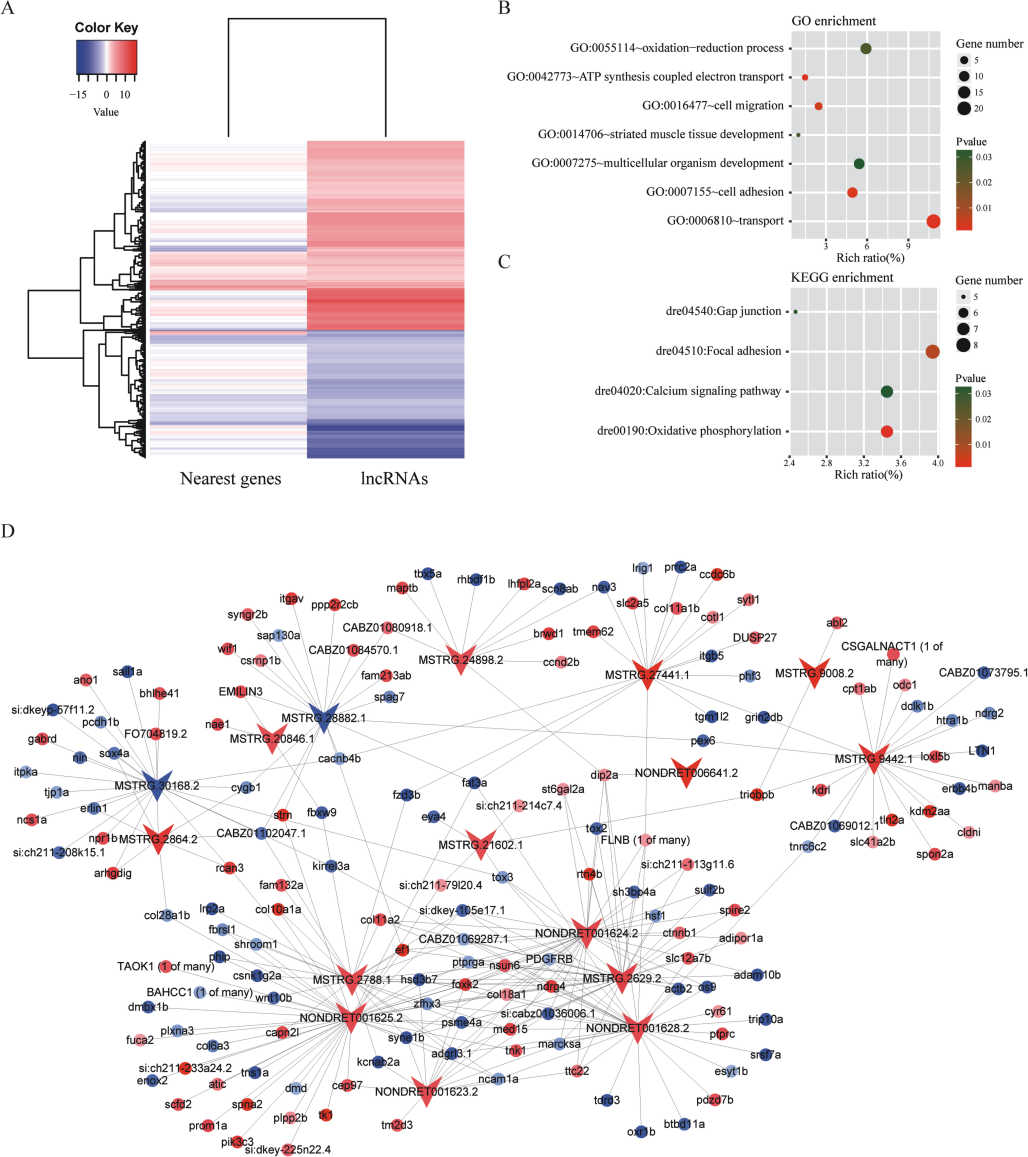
Predicted target genes of the differential expressed lncRNAs. (A) A heatmap was generated from the fold change values in the RNA-Seq data, and was used to visualize the expression patterns of the cold responsive lncRNAs and their neighboring genes after cold acclimation. (B-C) GO and KEGG enrichment analyses of the differentially expressed genes adjacent to the differentially expressed lncRNAs. The y-axis corresponds to KEGG pathway with a p-value ≤ 0.05, and the x-axis shows the enrichment ratio between the number of DE-genes and all unigenes enriched in a particular pathway. The color of the dot represents p value, and the size of the dot represents the number of DE-genes mapped to the reference pathways. (D) Regulatory network of 16 DE-lncRNAs with the lowest FDR, was built by Cytoscape 3.5. The triangles denote representative lncRNAs and the dots represent the trans-target genes. The colors represent log-transformed fold change. Blue: down-regulated; red: up-regulated.

To date there are still serious debates over the underlying mechanisms of transcriptional regulation by lncRNAs. Some studies suggested that lncRNAs regulate transcription of adjacent genes (in cis) [28–30]. Here the potential target genes of the DE-lncRNAs in cis were searched. The results showed 813 known protein-coding genes adjacent to 689 DE-lncRNAs, including upstream, downstream, and overlapping (S2 Table). Among these adjacent genes, 456 genes are less than 10 kb from the neighboring lncRNAs, and 765 are less than 100 kb from the nearest lncRNAs, and 230 genes were differentially expressed (FDR<0.05). Among the 230 differentially expressed genes (DE-genes) (Fig 3A), 170 genes showed the same expression trend with nearby lncRNAs, and only 60 genes showed the opposite expression trend with neighboring lncRNAs. GO and KEGG enrichment analyses of those differentially expressed genes showed significantly enriched GO terms including ATP synthesis coupled electron transport, cell adhesion, cell migration, oxidation-reduction process, striated muscle tissue development, and multicellular organism development (Fig 3B). Significantly enriched KEGG pathways included oxidative phosphorylation, focal adhesion, gap junction, calcium signaling pathway (Fig 3C). These results indicated that lncRNAs may participate in cold acclimation in zebrafish by regulation of electron transport, cell junction, oxidation-reduction, signal transduction, and muscle tissue development.

### Prediction of trans-target genes of the DE-lncRNAs

It has been reported that lncRNA can also regulate gene expression in trans (on other chromosomes or distal regions)[28], and lncRNA-mRNA interaction can affect mRNA level [31,32]. In this study, 20 DE-lncRNAs with the lowest FDR values were selected for prediction of their trans-target genes. RNAplex [25] was used for searching interactions between above mentioned lncRNAs and differentially expressed mRNAs (DE-mRNAs). It showed that 20 DE-lncRNAs have 1098 potential trans-target mRNAs (S3 Table).

The potential trans-target genes were used to perform GO and KEGG enrichment analyses (S4 Table). The top GO terms include organ development, cell adhesion, axon extension, and regulation of transcription from RNA polymerase II promoter. The most enriched pathways include Wnt signaling pathway, ECM-receptor interaction, one carbon pool by folate, and biosynthesis of antibiotics. To show the mode of interaction between lncRNAs and mRNAs, lncRNAs and mRNAs with a free energy less than -50 were used to draw an interaction network. According to this criterion, 16 lncRNAs corresponding to 176 target mRNAs were selected to build regulatory network. We noticed that one gene might be regulated by multiple lncRNAs and one lncRNA might also regulate multiple genes (Fig 3D).

### Verification of expression of lncRNAs and target genes by qRT-PCR

**Fig 4:**
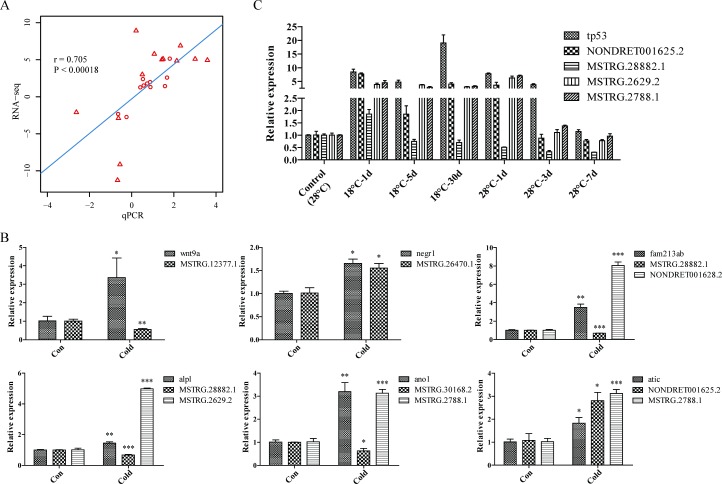
Comparison of the expression patterns of lncRNAs and cis or trans target protein-coding genes. Fold changes of gene expression detected by RNA-Seq were plotted against the data of qRT-PCR. The reference line indicates the linear relationship between the results of RNA-Seq and qRT-PCR. Triangles represent lncRNAs and circles represent protein-coding genes. (B) Relative expression levels of lncRNAs (MSTRG.12377.1, MSTRG.26470.1, MSTRG.28882.1, NONDRET001628.2, MSTRG.2629.2, MSTRG.30168.2, MSTRG.2788.1 and NONDRET001625.2) and their targets genes. (C) Dynamic changes of selected lncRNAs and *tp53* mRNA during cold acclimation and subsequent recovery process. Data are presented as means ± SD of three independent replicates. ACTB was used as the reference gene. *: p < 0.05, **: p < 0.01, ***: p < 0.001.

To validate the same or opposite expression trend between above mentioned lncRNAs and predicted target genes, qRT-PCR was applied to determine the levels of selected 13 lncRNAs and 11 protein-coding genes from control or cold acclimated ZF4 cells. The correlation between RNA-Seq and qPCR data was analyzed with Spearman’s rho test, and a highly statistical significance [r = 0.705, p < 0.00018] was observed. As shown in Fig 4A, the corresponding expression trends between these lncRNAs and their target genes are consistent with our RNA-Seq data. (Fig 4B) Genes *wnt9a* (Wingless-type MMTV integration site family, member 9A) and *negr1* (neuronal growth regulator 1) may be negatively or positively regulated by MSTRG.12377.1 and MSTRG.26470.1 respectively in cis. For each gene of *fam213ab* (family with sequence similarity 213, member Ab), *alpl* (alkaline phosphatase, liver/bone/kidney), *ano1* (anoctamin 1, calcium activated chloride channel) and *atic* (5-aminoimidazole-4-carboxamide ribonucleotide formyltransferase/IMP cyclohydrolase), which may be regulated by multiple lncRNAs in trans, we picked out two lncRNAs that had the lowest free energy interaction with them. The trends of expression of these lncRNAs and target genes were presented in Fig 4B and S1 Fig. Meanwhile, we investigated the dynamic expression changes of selected lncRNAs (1 annotated and 3 novel). The tp53 mRNA reported up-regulated in cold acclimated ZF4 cells served as a positive control[11]. ZF4 cells were exposed to cold stress for 1, 5, and 30 days and returned to normal culture temperature for 1, 3, and 7 days, then the expression of above RNAs was examined. As shown in Fig 4C, all RNAs increased 1 day after cold exposure and showed different trends thereafter, the expression of these RNAs on the 30^th^ day is consistent with the RNA-Seq data. We also noticed a recovery of RNA expression after the cells were returned to the normal temperature, NONDRET001625.2 and MSTRG.2629.2 showed no significantly difference compared with control cells after 3 days (P>0.05), while tp53 mRNA and MSTRG.2788.1 lncRNA returned to the normal levels after 7 days. But the expression of MSTRG.28882.1 still remained at a lower level after 7 days at 28°C, indicating different signaling pathways are involved in this process.

## Discussion

Pre-acclimation of fishes to moderate thermal stress can increase their tolerance to lethal thermal stress [9]. The current studies of mechanisms of cold acclimation of fish mainly involve genome, transcriptome, miRNA, DNA methylation, histone modification, and so on [9–12,33]. Cold stress responsive lncRNAs have been identified in plants and mammals [14,15], and tissue-specific and antibiotic toxicity responsive lncRNAs have been also reported in zebrafish [34,35]. So far, no detailed study of the roles of lncRNAs in cold acclimation of fish has been documented. In the present study, 8,363 novel lncRNAs were identified. most of them lay at intergenic regions and have less exons, shorter transcripts, lower expression levels compared with mRNAs. The expression of lncRNAs significantly decreased during cold acclimation. These characterizations are coincide with previous reports [1–3,14,34,35].

Totally 689 lncRNAs were differentially expressed under cold pressure in cold treated ZF4 cells compared with control cells. Among DE-lncRNAs, there are 74 or 61 lncRNAs expressed only in normal cultured or cold acclimated cells, respectively. It is interesting that the mRNAs from chromosome 4 showed obviously lower levels, while there is no significant difference between the expression levels of the lncRNAs from different chromosomes (Fig 2D). The low expression levels of mRNAs from chromosome 4 is also observed by reviewing the data from other reports about mRNA expression profiles of zebrafish tissues or cells, suggesting this expression pattern is not specific for ZF4 cells (S2 Fig)[36]. GO and KEGG enrichment analyses of the differentially expressed protein-coding genes (S5 Table) showed multiple biological processes and signal transduction pathways, such as cell junction, ion transport, muscle development, axon extension, P53 and FoxO signaling pathways are involved in cold acclimation of ZF4 cells. The results are similar with other *in vivo* studies [37,38].

Since lncRNAs can regulate their nearby genes, such as promoter upstream transcripts (PROMPTs) and enhancer RNA (in cis) [39]. Here GO and KEGG enrichment analyses of the differentially expressed cis-target genes showed involvement of electron transport, cell junction, oxidation-reduction, signal transduction, and muscle tissue development in cold acclimation. Previous studies also suggested that lncRNAs can interact with associated mRNAs via the formation of complementary hybrids (in trans) [31,32]. In this study 1,098 differentially expressed mRNAs were considered as trans-target mRNAs of top 20 DE-lncRNAs and were enriched in biological processes like organ development, cell adhesion, axon extension, regulation of transcription from RNA polymerase II promoter and KEGG pathways like Wnt signaling pathway, ECM-receptor interaction, one carbon pool by folate, biosynthesis of antibiotics. Many of these enriched biological processes and pathways have been reported associated with cold adaptation in previous studies [11,33,36]. These results indicated that lncRNAs might regulate these biological processes to participate in the regulation of cold acclimation.

Cold stress can lead to increased reactive oxygen species in fish tissues, while reducing reactive oxygen species will help fish adapt to low temperature environment [40]. *Fam213ab* which participates in oxidation-reduction process, may be regulated by lncRNAs MSTRG.28882.1 and NONDRET001628.2, and was up-regulated under cold stress (Fig 4B). Genes involved in folate metabolic pathway, like *alpl* and *atic* were also up-regulated under cold stress, and might be regulated by multiple lncRNAs in trans. Our previous study reported some genes involved in folate biosynthesis pathway were up-regulated and hypomethylated at promoter regions of genes under cold stress[11]. This indicated that both DNA methylation and lncRNAs might paly critical roles in folate metabolic pathway under cold pressure.

The present data shed new light on the role of lncRNAs in cold acclimation in fish. Further epigenetic regulation mechanisms of cold acclimation still need to be demonstrated in the future.

## Supporting information

S1 Fig Comparison of the expression patterns of lncRNAs and cis or trans target protein-coding genes. (A) Relative expression levels of lncRNAs (NONDRET008543.2, MSTRG.28882.1, MSTRG.9442.1, NONDRET001625.2, NONDRET001624.2, MSTRG.2629.2, NONDRET001628.2, MSTRG.21602.1) and their targets genes. Data are presented as means ± SD of three independent replicates. ACTB was used as the reference gene. *: p < 0.05, **: p < 0.01, ***: p < 0.001.
(TIF)

S2 Fig Distribution of mRNAs along each chromosome in zebrafish brain. Red and blue represent the log-transformed reads per kilobase per million mapped reads (RPKM) values of mRNAs in zebrafish brain under 28°C or 18°C, respectively(RNA-seq data from reference 39).
(TIF)

S1 Table List of primers used for qRT-PCR analysis. (XLSX)

S2 Table Expression information of DE-lncRNAs and nearby protein-coding genes. (XLSX)

S3 Table Information for the trans-target mRNAs of top 20 DE-lncRNAs. (XLSX)

S4 Table GO and KEGG enrichment analyses of trans-target genes. (XLSX)

S5 Table GO and KEGG enrichment analyses of total DE-genes. (XLSX)

## Abbreviations

lncRNA: long non-coding RNA
ZF4: zebrafish embryonic fibroblast cells
GO: Gene Ontology
KEGG: Kyoto Encyclopaedia of Genes and Genomes
FPKM: fragments per kilobase per million mapped reads
FDR: false discovery rate
DE: Differentially Expressed
