# Supplementary figures and images for: Characterization of lncRNAs involved in cold acclimation of zebrafish ZF4 cells
Source: PLoS One. 2018 Apr 10;13(4):e0195468. doi: 10.1371/journal.pone.0195468 (PMC5892903; doi:10.1371/journal.pone.0195468)

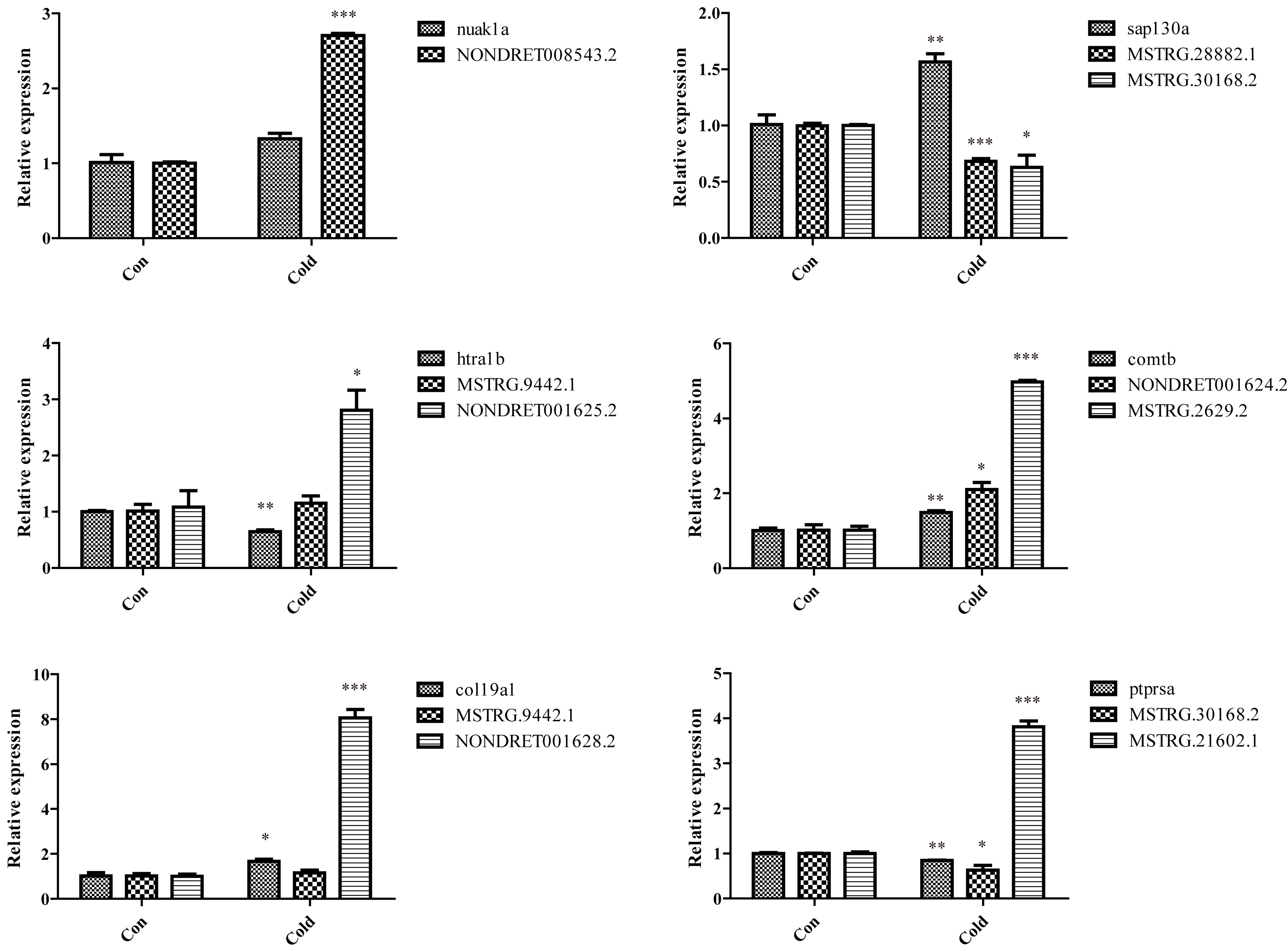

Supplement: S1 Fig — (A) Relative expression levels of lncRNAs (NONDRET008543.2, MSTRG.28882.1, MSTRG.9442.1, NONDRET001625.2, NONDRET001624.2, MSTRG.2629.2, NONDRET001628.2, MSTRG.21602.1) and their targets genes. Data are presented as means ± SD of three independent replicates. ACTB was used as the reference gene. *: p < 0.05, **: p < 0.01, ***: p < 0.001. (TIF) [file pone.0195468.s001.tif]

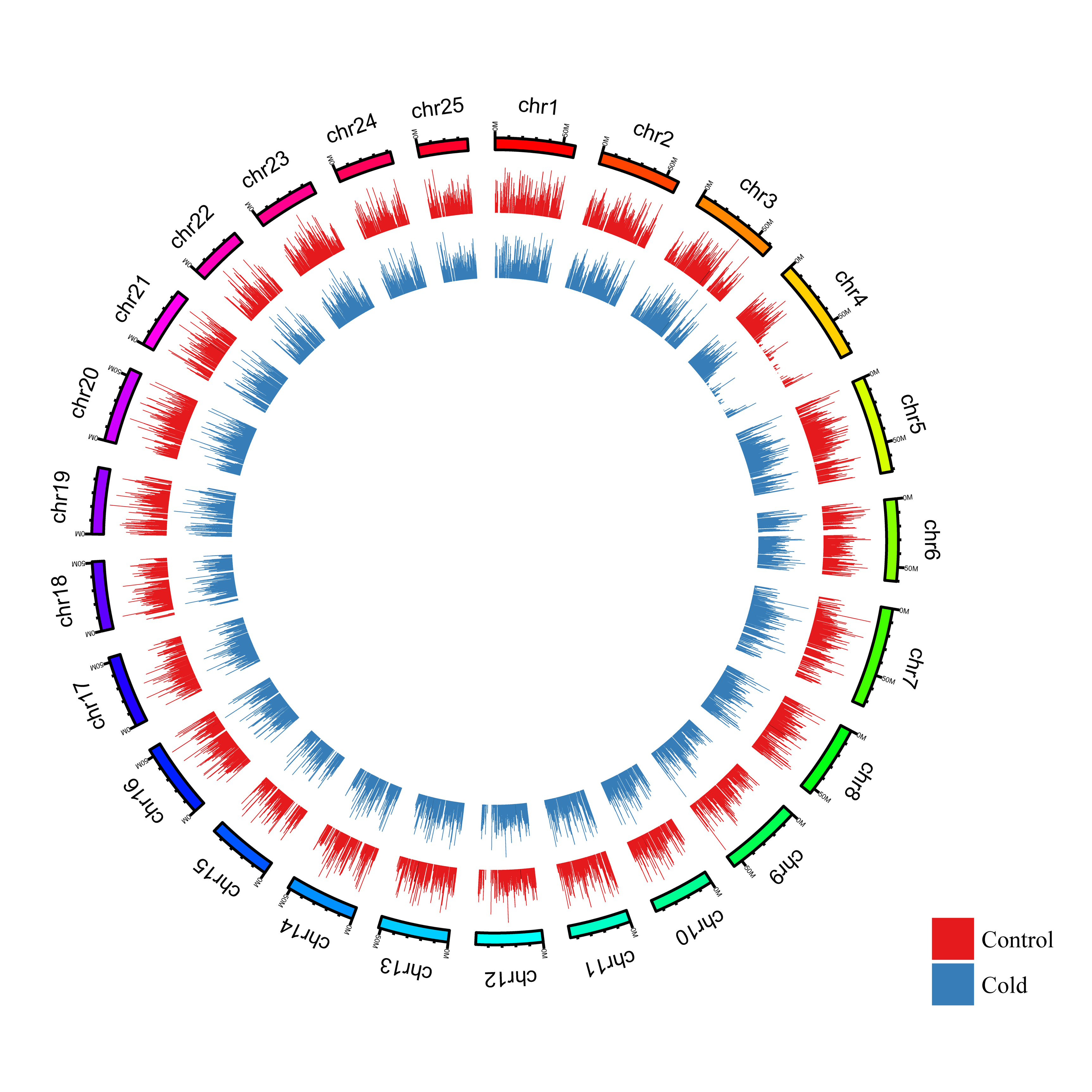

Supplement: S2 Fig — Red and blue represent the log-transformed reads per kilobase per million mapped reads (RPKM) values of mRNAs in zebrafish brain under 28°C or 18°C, respectively(RNA-seq data from reference 39). (TIF) [file pone.0195468.s002.tif]
